# Aldoxorubicin and Temozolomide combination in a xenograft mice model of human glioblastoma

**DOI:** 10.18632/oncotarget.26183

**Published:** 2018-10-09

**Authors:** Martina Da Ros, Anna Lisa Iorio, Veronica De Gregorio, Ornella Fantappiè, Giacomo Laffi, Maurizio de Martino, Claudio Pisano, Lorenzo Genitori, Iacopo Sardi

**Affiliations:** ^1^ Neuro-Oncology Unit, Department of Pediatric Oncology, Meyer Children’s Hospital, Florence, Italy; ^2^ Department of Experimental and Clinical Medicine, University of Florence, Florence, Italy; ^3^ BIOGEM Research Institute, Ariano Irpino, Italy

**Keywords:** aldoxorubicin, temozolomide, glioblastoma, malignant glioma, xenograft model

## Abstract

Glioblastoma Multiforme (GBM) is still an incurable disease. The front-line Temozolomide (TMZ)-based therapy suffers from poor efficacy, underlining the need of new therapies.

Preclinically, Aldoxorubicin (Aldox), a novel prodrug of Doxorubicin (Dox), has been successfully tested against GBM, encouraging the study of its association with other agents.

For the first time, we evaluated the effectiveness of Aldox combined to TMZ in preclinical models of GBM.

Our *in vitro* results demonstrated that the anti–glioma effect of Aldox was more marked than TMZ and their combination increased the killing effect of the anthracycline in TMZ-resistant GBM cells. Moreover, unlike Dox, Aldox was able to accumulate in P-glycoprotein (P-gp)-overexpressed cells due to a negative regulation of the P-gp function.

We also compared efficacy and safety of weekly administrations of Aldox (16 mg/kg), with or without TMZ (0.9 mg/kg, daily injections), in the U87 xenograft mouse model.

Aldox therapy induced a moderate tumor volume inhibition (TVI) and an increased survival rate (+12.5% *vs* vehicle). On the other hand, when combined to TMZ, Aldox caused a significant TVI (P=0.0175 *vs* vehicle) and delayed the mortality during the experimental period, although TVI and endpoint survival percentage (+37.5% *vs* vehicle) were not significantly different from TMZ alone.

Our preliminary data showed that Aldox exerts anti–glioma effects *in vitro* and *in vivo*. It also enhances its antitumor activity when combined with TMZ, resulting in a superior efficacy compared to the single agents, without adverse side effects.

## INTRODUCTION

There is a need for new approaches that may be able to achieve improved outcomes in Glioblastoma (GBM) patients.

In fact, despite multimodal approach of radiotherapy *plus* concurrent and adjuvant Temozolomide (TMZ), the prognosis for patients has not changed significantly for over ten years.

A shared issue among these malignancies that affects the efficacy of anticancer therapies involves the Blood-Brain Barrier (BBB); it represents a major hurdle for the delivery of drugs into the brain, contributing to the poor response of brain tumors to chemotherapy.

Doxorubicin (Dox) is an effective anti-glioma agent, but its clinical application is primarily limited by inability to cross the BBB [1].

At the present, various strategies have been emerged in order to increase the therapeutic use of the drug, including the incorporation in nanoscale carriers as liposomes, polymer and peptide/protein conjugates, polymeric micelles, nanoparticles [2].

An attractive new approach is a delivery system that exploits albumin as a drug carrier.

In 2002 Kratz and co-workers investigated antitumor efficacy and toxicity of four albumin-binding Dox prodrugs, demonstrating that especially the (6-maleimidocaproyl)hydrazone derivative of Dox (Aldoxorubicin (Aldox), formerly known as INNO-206, CytRx Corporation), selectively bound to the cysteine-34 position of endogenous albumin, was superior to the parent molecule Dox in a murine renal cell carcinoma model and in breast carcinoma xenograft models [3].

The study was taken further and the higher efficacy of Aldox compared to Dox was also demonstrated in an ovarian carcinoma model, in an orthotopic pancreatic cancer model [4], in a multiple myeloma model [5] and in a U87 human GBM xenograft model [6], with a 3- to 5-fold increase in the Maximum Tolerated Dose (MTD) [7].

In addition, preclinical studies have showed that Aldox is significantly less cardiotoxic than Dox [8] and, unlike Dox, it is able to penetrate the BBB [6], although it is still unclear why covalent binding to albumin allows Dox to bypass the export pumps at the BBB [9].

Of note that combination protocols with Aldox and Dox have produced hopeful results in solid malignancies as ovarian and pancreatic cancer [10, 11], encouraging the study of association of albumin-binding drugs with other anticancer agents.

For malignant GBM, TMZ is the only front-line treatment. However, no significant improvements are induced by TMZ treatment on overall survival and the combination therapy with other drugs, found to be effective against gliomas [12, 13], does not appear to improve response or survival of GBM patients [14].

Therefore, we first investigated the combinational effect of Aldox and TMZ against gliomas. Thus, we have studied the anti-glioma activity of this novel anthracycline, either alone or combined to TMZ, *in vitro* and determined its efficacy on tumor regression in a xenograft model of human GBM. Moreover, we have verified if the addition of TMZ affects the cellular uptake of Aldox, focusing on the activity of P-glycoprotein (P-gp), one of the most intensively studied multidrug transporters.

## RESULTS

### Effect of association of TMZ with Aldox on GBM growth and apoptosis *in vitro*

We investigated the responsiveness of three GBM cell lines (U87MG, A172 and T98G) to the killing effect of the Aldox (12 μM) and TMZ (100 μM and 200 μM) exposure and the co-treatment Aldox *plus* TMZ.

**Figure 1:**
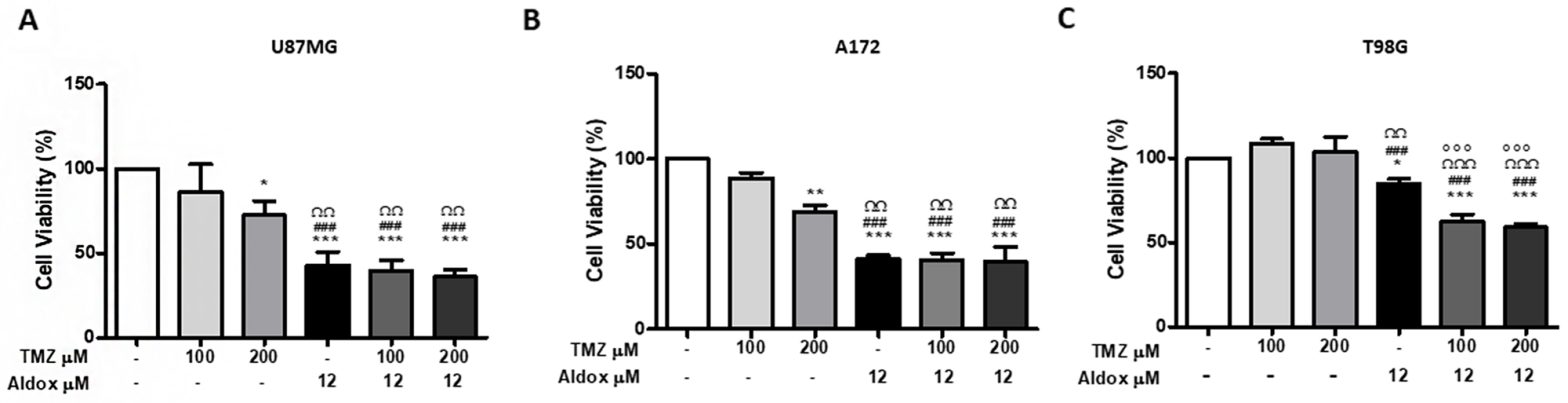
Cell viability of U87MG **(A)**, A172 **(B)** and T98G **(C)** cells treated with Aldox, TMZ and their combination at the indicated concentrations for 72 h by MTT assay. Results shown are the mean % viability ± S.D. of three independent experiments. ^*^P=<0.01; ^**^=P<0.001 ^***^=P<0.0001 *vs* untreated cells; ^###^=P<0.0001 *vs* TMZ 100 μM-treated cells; ^ΩΩ^P< 0.001; ^ΩΩΩ^P< 0.0001 *vs* TMZ 200 μM-treated cells; °°°P<0.0001 *vs* Aldox-treated cells; ANOVA one-way followed by Bonferroni post-hoc test.

Our data showed that TMZ at 100 μM was not toxic to all cell lines, while the higher concentration of 200 μM resulted to be toxic to U87MG and A172 (Figure 1A and 1B). T98G appeared to be TMZ resistant (Figure 1C).

In contrast, Aldox significantly inhibited the viability of all cell lines (^*^P<0.01; ^***^P<0.0001 *vs* untreated cells).

As shown in Figure 1A and 1B, the treatment with Aldox resulted in a cell viability of ∼50% of control in U87MG and A172, with a greater cytotoxicity compared to TMZ (^###^P<0.0001 *vs* TMZ 100 μM-treated cells; ^ΩΩ^P< 0.001 *vs* TMZ 200 μM-treated cells), while T98G turned out to be more resistant with a cell viability of ∼80% of control (Figure 1C).

Even more interesting, the killing effect of the anthracycline against T98G was significantly higher in the presence of both low and high TMZ (°°°P<0.0001 *vs* Aldox-treated cells) (Figure 1C).

We also performed a TUNEL assay to investigate the pro-apoptotic effect of Aldox (12 μM) and TMZ (100 μM and 200 μM), as single therapy and combined treatment, on GBM cells.

**Figure 2:**
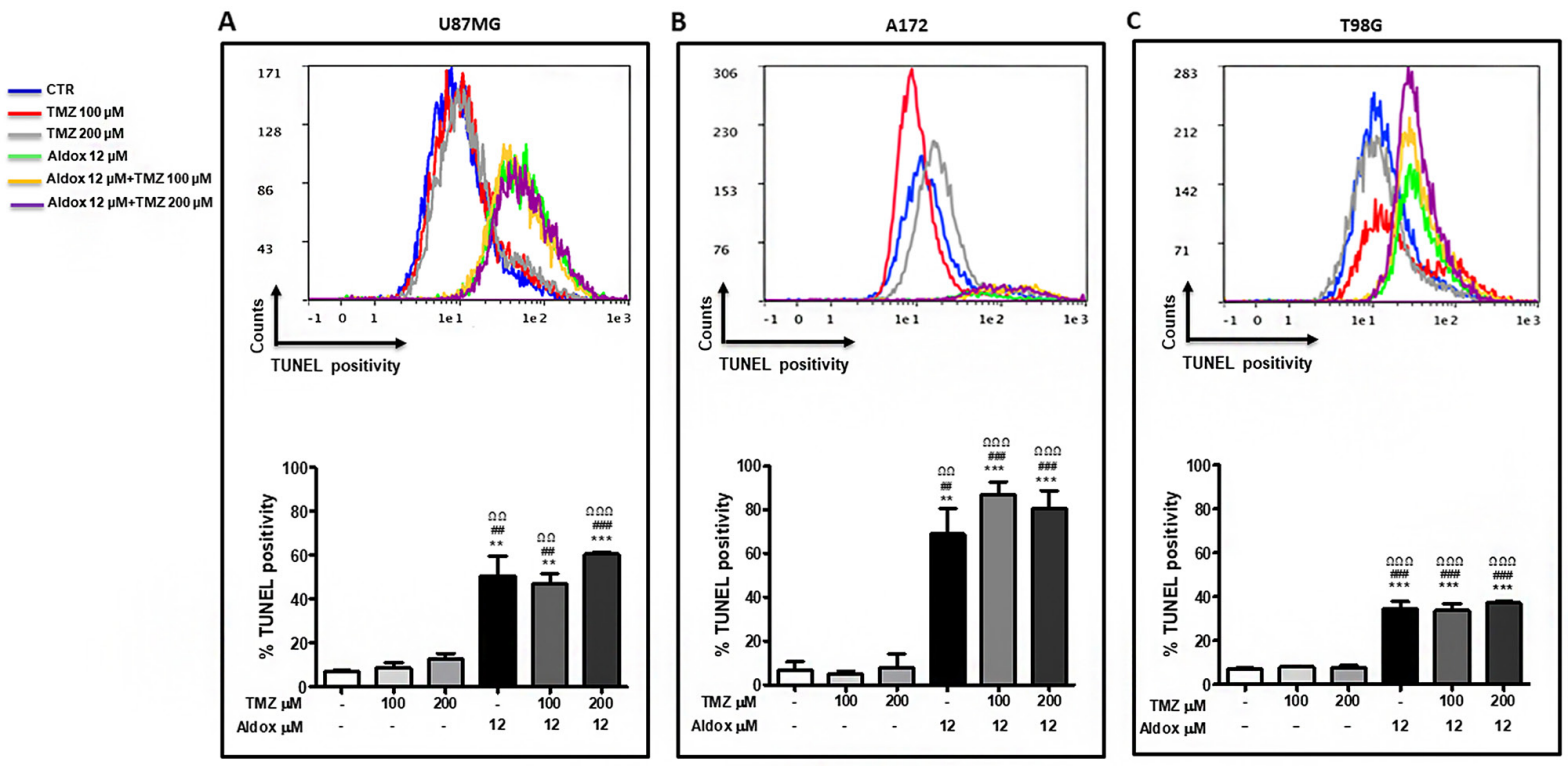
Pro-apoptotic effect of Aldox, TMZ and their combination (30 h) on U87MG **(A)**, A172 **(B)** and T98G **(C)** cells. Upper panel: qualitative representation of TUNEL positivity; lower panel: quantitative representation of TUNEL positivity (mean % TUNEL positivity ± S.D) ^**^P=<0.001; ^***^=P<0.0001 *vs* untreated cells; ^##^=P<0.001; ^###^=P<0.0001 *vs* TMZ 100 μM-treated cells; ^ΩΩ^P< 0.001; ^ΩΩΩ^P< 0.0001 *vs* TMZ 200 μM-treated cells; ANOVA one-way followed by Bonferroni post-hoc test. Experiments performed two times in triplicate.

Flow cytometry demonstrated that the rate of apoptosis was very lower in untreated-, 100 μM- and 200 μM TMZ groups compared with Aldox and Aldox *plus* TMZ groups in all cell lines (^**^P<0.001; ^***^P<0.0001 *vs* untreated cells; ^##^P<0.001; ^###^P<0.0001 *vs* TMZ 100 μM-treated cells; ^ΩΩ^P< 0.001; ^ΩΩΩ^P< 0.001 *vs* TMZ 200 μM-treated cells) (up to 8000 acquired events for each sample). Consistent with the results of cell viability assay, Aldox-based treatment induced apoptosis in more than 50% of U87MG (Figure 2A) and its killing effect against A172 was reflected in the low number of acquired cells, mostly apoptotic cells (Figure 2B).

On the other hand, the apoptotic rate dropped to ∼30% in T98G cells (Figure 2C).

Judging from these data, we further investigated the pro-apoptotic effect of Aldox, either alone and combined to TMZ, in T98G cells prolonging treatments for 48h. At this time point, untreated-, TMZ- and Aldox-treated samples have been successfully acquired and analyzed, confirming the pro-apoptotic effect of Aldox only, while the reliable cytometric evaluation of apoptosis in Aldox-TMZ-treated samples was not possible due to the unsatisfactory number of acquired events (< 1000 events) (data not shown).

### Effects of TMZ and Aldox on the P-gp function

We studied the effects of TMZ and Aldox on the P-gp ATPase activity, detected as a luciferase-generated luminescent signal, and measured in terms of change in luminescence and ATP consumed by P-gp.

**Figure 3:**
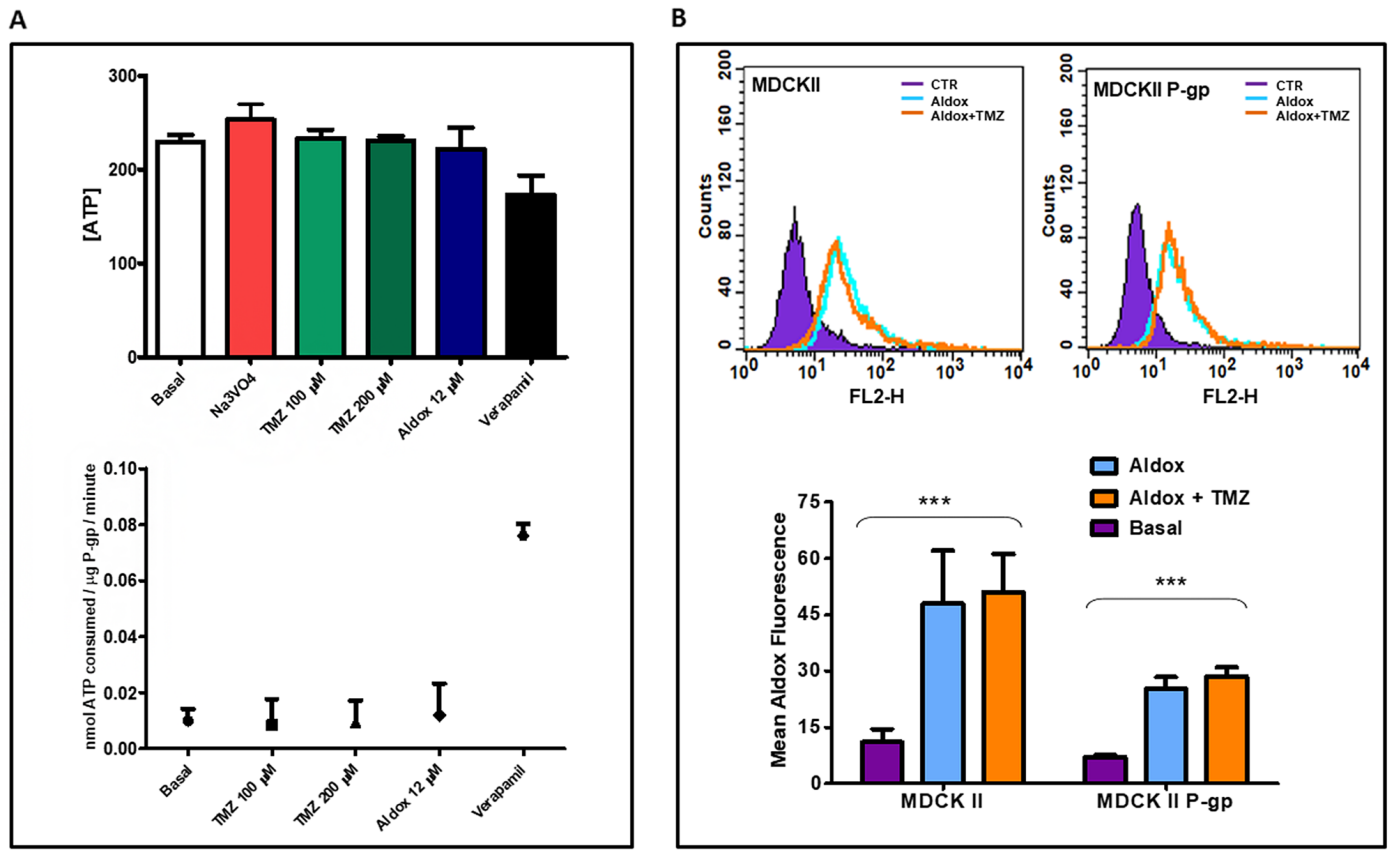
**(A)** Drug stimulated P-gp ATPase activity was estimated by Pgp-GLOassay system and measured in terms of both change in luminescence, that reflects the ATP concentration (upper panel), and ATP consumption (nmol ATP consumed/mg P-gp/minute) (lower panel), that reflects the P-gp ATPase activity. Data expressed as mean ± SD. Experiments performed two times in triplicate. **(B)** The uptake of Aldox, with or without TMZ, was evaluated in MDCKII parental and P-gp transfected cells by flow cytometry, using the FL2-H channel. Upper panel: qualitative representation of flow cytometric detection of Aldox uptake; lower panel: quantitative representation of Aldox uptake (Mean Aldox Fluorescence ± S.D), ^***^=P<0.0001 *vs* untreated samples; ANOVA one-way followed by Bonferroni post-hoc test. Results representative of two independent experiments in triplicate.

As shown in the upper panel of Figure 3A, samples treated with TMZ and Aldox contained a higher amount of ATP compared to Verapamil sample (^**^P=0.0011), reflecting a reduced consumption of ATP by P-gp (lower panel, Figure 3A).

The rate of ATP consumption due to Basal P-gp ATPase activity (calculated as difference in luminescent signal between Na_3_VO_4_-treated and untreated samples) was 0.010±0.004 while Verapamil stimulated the ATPase activity of P-gp with >6-fold stimulation of the basal activity.

In the presence of TMZ (both low and high doses) and Aldox, P-gp consumed ATP equal to or less than basal control (no statistically significant differences) and Verapamil-positive control (^**^P=0.0013), indicating that these drugs were able to adversely affect the P-gp ATPase activity

To study the possible P-gp-mediated TMZ-Aldox interaction, we also performed uptake experiments on MDCKII, parental and P-gp transfected cells, taking advantage of Aldox autofluorescence (upper panel, Figure 3B).

As expected, the mean fluorescence intensity of Aldox in MDCKII parental cells was superior to that of MDCKII P-gp cells (47.93 *vs* 25.30). However, compared to untreated P-gp trasfected cells (7.13), the same samples exposed to Aldox, as well as to Aldox-TMZ, contained a significant higher amount of the anthracycline (25.30, ^***^P<0.0001) (lower panel, Figure 3B), indicating its inhibitory effect on the efflux pump activity.

### The *in vivo* anti–glioma effects of Aldox-TMZ combined therapy

We evaluated Aldox at 16 mg/kg (50% MTD) once weekly for up 5 weeks alone and in combination with TMZ (0.9 mg/kg, daily administrations).

**Figure 4:**
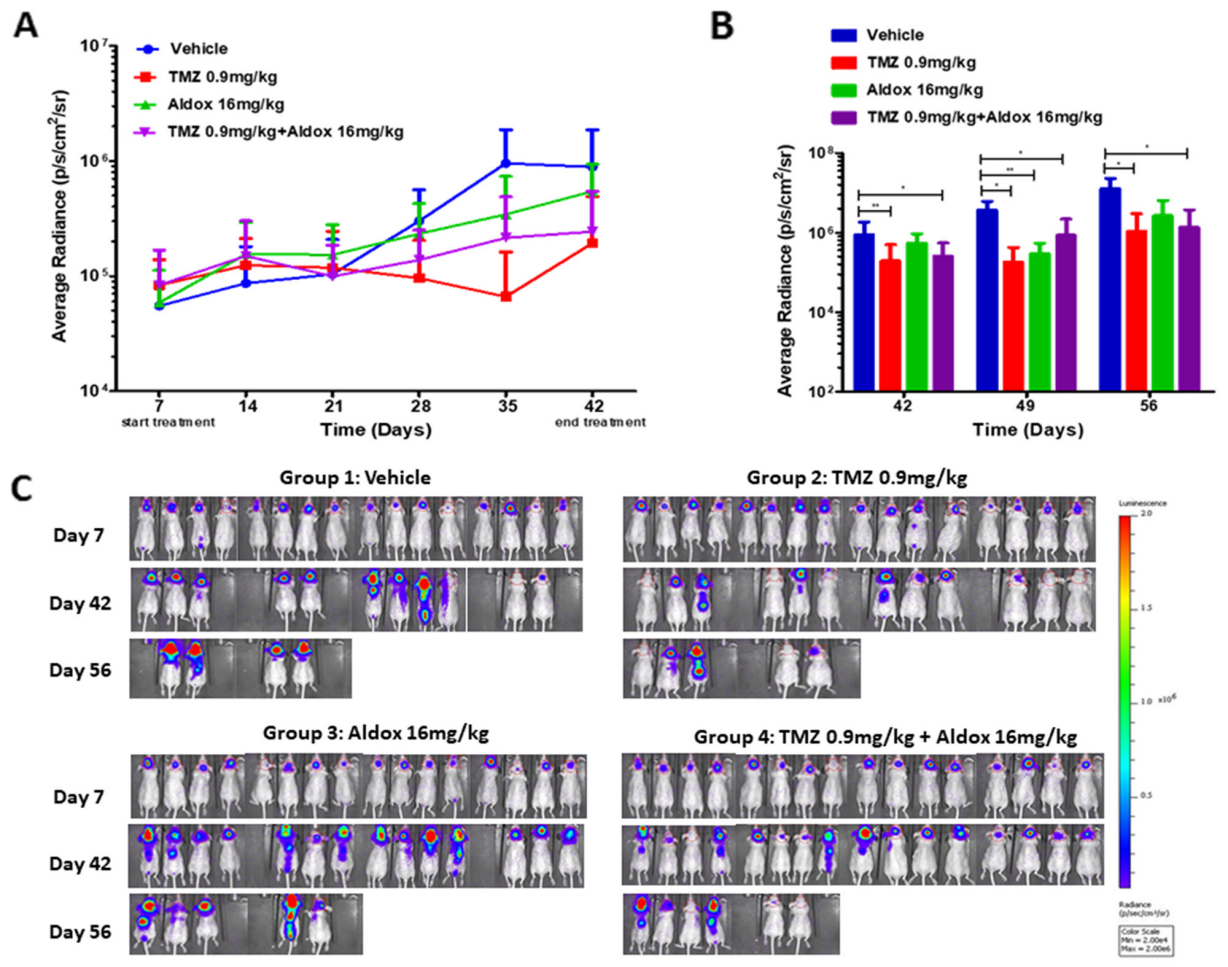
**(A)** BLI signals throughout the experimental period, distinctly for group of treatment and day from injection (from day 7 to day 42). **(B)** Average IVIS values between experimental groups at day +42, +49 and +56. Data expressed as mean ± SD. Outliers were removed by the ROUT test (Q=1%). Statistical analysis was performed with Mann-Whitney *U*-Test (^**^=P<0.01; ^*^=P<0.05). **(C)** Images of BLI acquisition of brain tumor on day 7 (start of treatments), day 42 (end of treatments) and day 56 (15 days after the end of treatments). The colorimetric scale represents the range of radiance values (red=highest value; blu=lowest value) which translates to tumor growth.

At the end of treatments (from day 7 to day 42), U87–bearing mice treated with Aldox alone showed less tumor compared to vehicle, with a bioluminescence (BLI) average radiance 2-fold lower than untreated animals. On the contrary, the association Aldox-TMZ induced a remarkable decrease of tumor growth, with an average BLI 4-fold lower than vehicle, which did not differ from the effect obtained by the administration of TMZ alone (Figure 4A).

On day 42, the mean of IVIS data in TMZ-treated mice was 1.93E+05 (P=0.0056 *vs* vehicle) and in those treated with Aldox *plus* TMZ was 2.43E+05 (P=0.0175 *vs* vehicle), to indicate a significant reduction of tumor growth compared to vehicle (8,87E+05) (Figure 4B).

In addition, by day 56 (15 days after the end of treatments), the tumor sizes in Aldox-, TMZ- and Aldox *plus* TMZ- treated mice were ∼4-fold, ∼11-fold and ∼9-fold smaller when compared to controls. The treatment with Aldox alone induced a reduction trend in tumor growth, although not statistically significant compared to vehicle (P=0.0635), while its association with TMZ caused a tumor volume inhibition (TVI) of about 90% (P=0.0190 *vs* vehicle) (Figure 4B). This effect did not significantly differ from that obtained by the administration of TMZ alone (TVI 92%, P=0.0317 *vs* vehicle).

In the plot shown in Figure 4B, the BLI values measured after day +56 have been omitted due to a high mortality, especially in vehicle and Aldox-treated mice.

The images of BLI acquisition of brain tumors on days + 7, + 42 and + 56 are shown in Figure 4C.

### Effect of Aldox-TMZ combined therapy on survival time of mice bearing GBM tumors

**Figure 5:**
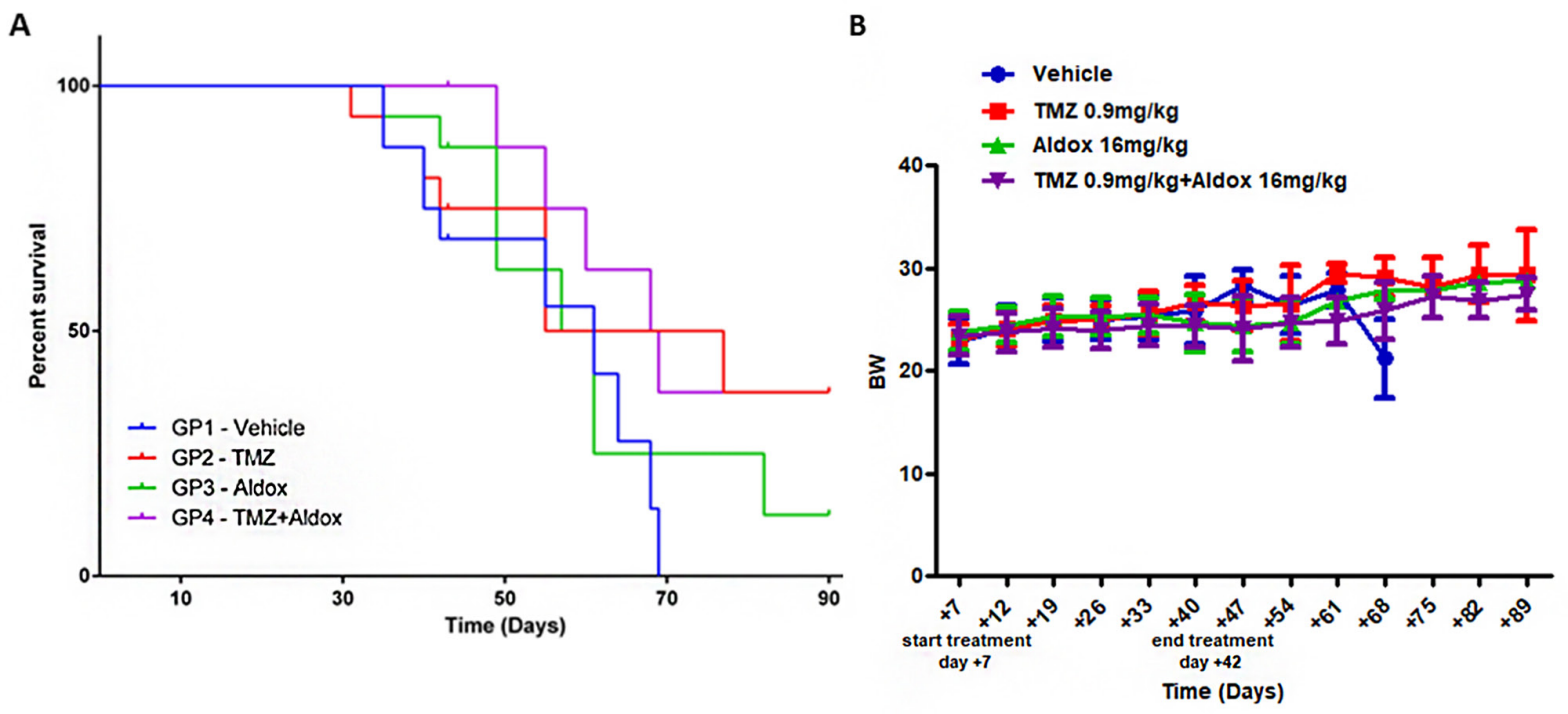
**(A)** Survival curves were compared between mice bearing GBM tumors, treated with vehicle, TMZ, Aldox or Aldox *plus* TMZ. Analysis was performed with Kaplan–Meier test. **(B)** BW measurements were used to evaluate the drug-related side effects. No statistical significant differences were reported between all experimental groups. Data expressed as mean ± SD.

The effect of a combination therapy schedule of Aldox and TMZ on mice survival is shown in Figure 5A. The study results demonstrated that Aldox improved the survival of mice bearing U87-tumors (+12,5% by day 90) when compared to vehicle-treated group who died within 69 days of tumor cell implantation; on the other hand, its association with TMZ leaded to an increase of survival time with a rate of +37,5% (*vs* vehicle).

Most interestingly, although the endpoint survival percentage was equal between TMZ- and Aldox *plus* TMZ-treated mice (37,5%), the combination therapy delayed the mortality observed during the experimental period (until day 90) when compared to TMZ alone, without side effects which were judged by body weight (BW) variations (Figure 5B).

## DISCUSSION

Combination regimens of targeted and immunotherapies with TMZ have been tested in several clinical trials for high grade glioma patients; currently, none of these studies has yet reached a significant clinical benefit in terms of overall survival or progression free survival [13, 14]. Powerful anticancer agents as Dox had poor impact on brain tumors [15] due to various resistance mechanisms, including the active efflux of anticancer drugs mediated by BBB transporters (specially P-gp). On the other hand, agents as TMZ, which has a good BBB penetration, have to be administered in high systemic doses to achieve therapeutic brain levels because of short half-life in plasma [16].

Albumin has been recently described “as an attractive candidate for targeted intracellular delivery of drugs attached by covalent conjugation, genetic fusions, association or ligand-mediated association” [17].

Among albumin-based systems in clinical trials, Aldox (the albumin-binding Dox, also known as INNO-206 or DOXO-EMCH) has been very successful, exhibiting a good profile in a phase 3 study in soft tissue sarcoma and in phase 2 studies in small cell lung cancer, Kaposi’s sarcoma and GBM. It has also been tested in combination therapies to treat solid malignancies [10, 11, 18], encouraging the study of association of albumin-binding drugs with other anticancer agents.

This study explored the preclinical effectiveness of a combination therapy schedule of TMZ and Aldox in comparison to the effect of the single agents against gliomas *in vitro* and *in vivo*.

Based on cell culture experiments, well-known TMZ sensitive U87MG and A172 (IC50 < 200μM) [19, 20] showed reduced viability (Figure 1A and 1B) and high apoptotic rate (Figure 2A and 2B) when treated with Aldox 12 μM (IC50 value at 72h), without significant difference among Aldox-TMZ combined treatment and Aldox therapy alone. On the other hand, this novel anthracycline induced moderate cytotoxic and apoptotic effects (Figure 1C and 2C) on the TMZ-resistant T98G [19, 20], that we have previously demonstrated to be also a Dox-resistant cell line [21]. The cytotoxicity became remarkable with the co-exposure to TMZ, resulting in a cell viability of 50% of control and in a significant higher killing effect compared to Aldox alone (Figure 1C). Unfortunately, flow cytometry performed after 48h of treatments did not allow achieving a reliable evaluation of apoptosis in Aldox-TMZ groups due to the unsatisfactory number of T98G acquired cells (data not shown). This finding was consistent with the cellular suffering observed microscopically and it was further reflected in the cytotoxicity data recorded at 72h.

We also evaluated the effect of Aldox on P-gp by studying its ATPase activity. Some studies have shown a synergistic effect on glioma cells for TMZ in association with other chemotherapeutic agents, identifying the direct negative modulation of P-gp by TMZ as one of the possible mechanisms [12, 22]. Our data demonstrated an increased ATP concentration (upper panel, Figure 3A), reflecting a reduced activity by P-gp (lower panel, Figure 3A), with addition of Aldox as well as of TMZ (no difference among low and high doses). We then characterized the intracellular uptake of Aldox and analyzed if there was any change after the co-exposure to a low dose of TMZ. Based on the results, the co-exposure to TMZ did not significantly improve the uptake of Aldox, itself able to accumulate in P-gp-overexpressed cells (upper panel, Figure 3B) due to a negative regulation of the P-gp function. As shown in Figure 3B (lower panel), the mean fluorescence intensity of Aldox in MDCKII parental cells was superior to that of MDCKII P-gp cells; however, compared to untreated P-gp trasfected cells, the same samples exposed to Aldox contained a higher amount of the anthracycline, unlike the previously observed with Dox [23].

Finally, to determine whether our *in vitro* findings would translate into anti–glioma effect *in vivo*, we evaluated the Aldox activity in combination with TMZ on tumor regression in a xenograft model of human GBM. U87-luc cells were injected in the right lobe of the brain of *Foxn1* nude mice, which received vehicle (saline), weekly intravenous injections of Aldox (16 mg/kg), daily administrations of TMZ (0.9 mg/kg) or their combination for up 5 weeks.

In our U87-xenograft model, the Aldox therapy produced a moderate TVI; on the contrary, its association with TMZ leaded to a remarkable reduction of tumor growth, without significant difference from the effect obtained by the administration of TMZ alone.

Whereas the anti-glioma effect of Aldox was observable at the end of pharmacological treatment (from day 35), it is important to note that the combined therapy with TMZ was effective from early injections (Figure 4A) and the combination of both agents proved still superior with longer follow up. From day 42 to day 56 (Figure 4B), the Aldox-TMZ schedule maintained a reduction trend in tumor growth with a TVI of 90% (P=0.0190 *vs* vehicle) and tumor sizes ∼2-fold smaller than Aldox treated-group (Figure 4C).

Our study confirmed also an improved survival time in Aldox-treated animals compared to vehicle group (+12.5% by day 90) (Figure 5A), as already shown by Marrero’s group [6]. Long-term efficacy and toxicity profile, which was judged by BW variations (Figure 5B), were similar to those obtained with daily administrations of TMZ 0.9 mg/kg, whose anti-glioma effects have been recently demonstrated in the same orthotopic xenograft model [24].

Most importantly, the association with TMZ tripled the survival rate (+37.5% *vs* vehicle) and, although the endpoint survival percentage was equal between TMZ- and Aldox *plus* TMZ-treated mice, the combined therapy delayed the mortality during the experimental period (until day 90) when compared to TMZ alone.

Intriguingly, the Aldox dose of 16 mg/kg (48 mg/m^2^ [25]) is 4-fold lower than the recommended dose of 200 mg/m^2^ in clinical trials [6], and a cumulative TMZ dose of 26 mg/kg(78 mg/m^2^ [25]) is far below the dose of 75 mg/m^2^/day used in oncology practice [26], resulting in reduced manifestations of drug-related side effects.

Taken together, the data obtained in our preclinical model suggest that the association of Aldox and TMZ has a better therapeutic efficacy for gliomas comparing to the single agents and provide a strong rationale for testing the anticancer activity of this combination in clinical studies in patients with high-grade gliomas.

## MATERIALS AND METHODS

### Chemicals and drugs

INNO-206 (Aldox) (MedChem Express, 10 mg and 100 mg) and TMZ (Sigma Aldrich, 25 mg) were dissolved in dimethyl sulfoxide and physiological saline solution for *in vitro* and *in vivo* test, respectively.

*In vitro* treatments were performed adjusting the drugs with culture medium to the final concentration of 100 μM and 200 μM for TMZ and 12 μM for Aldox at the time of treatment.

For *in vivo* experiments, TMZ and Aldox were prepared on each day of injection at the concentration of 0.9 mg/kg and 16 mg/kg per injection, then administered per os (OS) and intravenously (IV, tail vein), respectively.

### Cell lines

Human GBM cell lines U87MG, T98G, A172 (American Type Culture Collection, ATCC) and parental and P-gp transfected Madin-Darby canine kidney epithelial cells (MDCKII, Netherlands Cancer Institute, Amsterdam) were cultured at 37°C in the presence of 5% CO_2_.

Dulbecco’s modified Eagle’s medium with 10% fetal bovine serum was used for all cell lines except for U87MG and T98G, maintained in Essential medium containing 10% serum.

For establishing the intracranial xenograft GBM tumor, U87MG-luc2 cells (PerkinElmer Italia S.P.A., Italy), expressing a luciferase reporter gene, were maintained in M-199 medium until the time of implantation.

### Chemotherapeutic sensitivity assay

U87MG (4×10^4^/well), A172 (4×10^4^/well) and T98G (3×10^4^/well) cells were plated in 24-well plates in triplicate, allowed to attach for 24h and then exposed to TMZ, Aldox and their combination at the indicated concentrations for 72h.

MTT Cell Proliferation Assay (Cayman Chemical, USA) was performed to study cell viability, following the manufacturer’s instructions.

A MULTISKAN FC (Thermo Scientific) microplate reader at a test wavelength of 550 nm was used to read absorbance values.

## TUNEL

U87MG (32 × 10^4^ cells/dish 60) and A172 (32 × 10^4^ cells/ dish 60) cells were cultured for 24h and treated with TMZ, Aldox and their combination for 30h; T98G cells (20 × 10^4^ cells/ dish 60) were exposed to drugs for 30h and 48h.

Samples were then fixed, blocked, permeabilized and TUNEL (terminal deoxynucleotidyl transferase–mediated dUTP nick end labeling) was performed by *in situ* cell death detection kit fluorescein (Roche), following the manufacturer’s instructions.

Apoptosis was analyzed by a flow cytometer (MACSQuant^®^ Analyzer 10, Miltenyi Biotech), using the FlowLogic Software (Inivai Technologies).

All experiments were performed two times in triplicate.

### P-gp ATPase activity

Drug stimulated P-gp ATPase activity was estimated by Pgp-Glo Assay System (Promega, Madison, WI) and measured in the presence of TMZ and Aldox, using the GloMax^®^ 96 Microplate Luminometer (Promega).

Experimental studies and data analysis were performed according to manufacturer’s guidelines, as previously described [24].

Briefly, in a 96 well plate recombinant human P-gp was incubated with P-gp-Glo assay buffer™ (control), verapamil (positive control), sodium orthovanadate (Na_3_VO_4_, P-gp ATPase inhibitor), TMZ 100 μM, TMZ 200 μM and Aldox 12 μM. A luciferase reaction mixture initiated an ATP-dependent luminescence reaction, then measured in terms of both change in luminescence and ATPase activity by interpolation from an ATP standard curve.

Luminescence changed in direct proportion to ATP concentration and the rate of ATP consumption (nmol ATP consumed/mg P-gp/minute) by P-gp was determined as difference between the amount of ATP in absence and presence of Na_3_VO_4_ (Basal P-gp ATPase activity).

All experiments were performed two times in triplicate.

### Intracellular Aldox accumulation

The cellular uptake of Aldox was analyzed using a FACScan flow cytometer (Becton Dickinson, Mountain View, CA, USA), equipped with a 488 nm argon laser. The exponentially growing parental and P-gp transfected MDCKII cells were treated with indicated concentrations of TMZ, Aldox and their combination at 37°C for 2 h, according to the previously described method [23].

Aldox fluorescence was determined in 8000 events for each sample.

### Xenograft study

GBM tumors were induced in 80 *Foxn1* nude male mice, 6 weeks-old, by implantation of 3x10^5^ U87MG-luc cells in the right lobe of the brain.

One week after tumor implantation, 64 animals were randomly assigned to four groups (16 mice/group) to be treated with different drugs, as follow:

- Group 1 (vehicle): weekly intravenous (IV) administration of physiologic solution, i.e., 7, 14, 21, 28, 35 days.

- Group 2: daily oral (OS) administration of TMZ 0.9 mg/kg, from day 7 to day 35.

- Group 3: weekly administration of Aldox 16 mg/kg (50% MTD), IV, i.e., 7, 14, 21, 28, 35 days.

- Group 4: TMZ-Aldox combination for up five weeks.

At the end of treatments (day 42, one week after the last injection), 32 animals were sacrified by CO_2_ inhalation; the remaining mice (8 animals/group) were observed for mortality until the 90th day of the study.

BW measurements were carried out 2 times a week and BLI imaging acquisition (IVIS spectrum image system, Perkin Elmer) were performed at day 0 (immediately after tumor implantation), at day 3 and then weekly until the end of the experiment (day 90).

A BW loss ≥ 15% has been considered as sign of suffering, involving the mouse sacrifice.

All experiments were conducted in accordance with the Institutional Animal Care and Use Committee guidelines.

The state of health of animals has been observed daily; if clinical signs showed clear suffering status, mice have been sacrificed under the veterinary and study director approval.

### Statistical analysis

Data shown represent mean ± SD. Analyses were carried out using GraphPad Prism 5.

Radiance data were analyzed by Mann-Whitney *U*-test at the indicated time points. The ROUT test (Q=1%) was used to remove outliers.

Survival analysis was performed by Kaplan–Meier curves and the log-rank test.

All *in vitro* experiments were repeated two or three times in triplicate and one-way ANOVA with a post hoc analysis by the Bonferroni *t*-test was used for multiple comparisons.

Significance was determined at P<0.05.

## Abbreviations

GBM: Glioblastoma Multiforme
TMZ: Temozolomide
Aldox: Aldoxorubicin
Dox: Doxorubicin
P-gp: P-glycoprotein
TVI: Tumor Volume Inhibition
BBB: Blood Brain Barrier
MTD: Maximum Tolerated Dose
BLI: Bioluminescence
BW: Body Weight
